# Role of milk glycome in prevention, treatment, and recovery of COVID-19

**DOI:** 10.3389/fnut.2022.1033779

**Published:** 2022-11-08

**Authors:** Merve Kaplan, Arif Sercan Şahutoğlu, Sümeyye Sarıtaş, Hatice Duman, Ayşenur Arslan, Burcu Pekdemir, Sercan Karav

**Affiliations:** ^1^Department of Molecular Biology and Genetics, Çanakkale Onsekiz Mart University, Çanakkale, Turkey; ^2^Department of Chemistry, Çanakkale Onsekiz Mart University, Çanakkale, Turkey

**Keywords:** glycosylation, human milk oligosaccharides, glycans, whey protein, COVID-19, SARS-CoV-2, antiviral activity

## Abstract

Milk contains all essential macro and micro-nutrients for the development of the newborn. Its high therapeutic and antimicrobial content provides an important function for the prevention, treatment, and recovery of certain diseases throughout life. The bioactive components found in milk are mostly decorated with glycans, which provide proper formation and modulate the biological functions of glycosylated compounds. The glycome of milk consists of free glycans, glycolipids, and *N-* and *O-* glycosylated proteins. Recent studies have shown that both free glycans and glycan-containing molecules have antiviral characteristics based on different mechanisms such as signaling, microbiome modulation, natural decoy strategy, and immunomodulatory action. In this review, we discuss the recent clinical studies and potential mechanisms of free and conjugated glycans' role in the prevention, treatment, and recovery of COVID-19.

## Introduction

Severe Acute Respiratory Syndrome Coronavirus-2 (SARS-CoV-2) is a single-stranded enveloped RNA virus that belongs to the beta coronavirus family. It was initially identified in humans in late 2019, which immediately caused a serious outbreak all over the world. The World Health Organization (WHO) reported the SARS-CoV-2 outbreak in the city of Wuhan, China in January 2020. Subsequently, WHO named this novel disease COVID-19 (1). Transmission of COVID-19 is usually through the respiratory system thus, the transmission commonly takes place by droplets spreading by coughing, inhaling, or sneezing aerosols (2). COVID-19-infected people show a variety of clinical symptoms ranging from cold-like symptoms to more severe ones that may cause pneumonia, coma, and death (3). According to the WHO reports, about 500 million people in the world have been affected by COVID-19 and more than 6 million deaths were recorded as of 01 July 2022. Many studies have been conducted to discover a preventive measure or treatment for COVID-19 since the beginning. Regarding vaccine studies, distinct types of vaccines such as messenger RNA (mRNA) based, DNA-based, viral-vector based, protein-based, and inactivated ones have been developed to combat the COVID-19 (4). Parameters including safety and efficacy have been considered during the treatment (5). However, the mutation tendency of the virus still results in the formation of different variants of SARS-CoV-2, which causes challenges in the production of an effective vaccine and treatment for COVID-19. Even though vaccines have been developed based on spike protein structure to be more effective, potential mutations on spike proteins create the same risk for those vaccines (6). Therefore, it is undoubtedly needed to seek new alternatives for treatment methods of COVID-19. Many researchers are still looking for new and effective treatments that may be effective to deal with the COVID-19 pandemic, leading many scientists to investigate the antiviral and anti-SARS-CoV-2 properties of bioactive components derived from milk.

Mammalian milk is recognized as the major source of immunity for newborns since it contains various bioactive components such as free and conjugated oligosaccharides (7). Glycoconjugates and their glycans counterparts are known to have antiviral activities against different viruses such as ranging adenoviruses, noroviruses, human immunodeficiency virus (HIV) etc., (8, 9). Lactoferrin, a glycoprotein in mammal milk, displays an antiviral effect on different types of viruses including SARS-CoV-2 which is the main focus of this review (10). Additionally, milk also contains other glycoconjugates that are potentially involved in antiviral mechanisms and human immunity. A key benefit of these proteins is their potential to prevent serious viral infections (11–13). Besides, free oligosaccharides have also been considered as a treatment strategy due to their antiviral activity. Especially, human milk oligosaccharides (HMOs), which are non-nutritional, complex carbohydrates, that may be used in applications to treat SARS-CoV-2 due to their functions such as receptor decoying, immunomodulating, prebiotics, as well as signaling agents (14). This review comprehensively summarizes the antiviral effects of milk oligosaccharides and glycoconjugates as well as describes the potential mechanisms of their actions upon COVID-19.

## Glycoproteins

**Figure 1:**
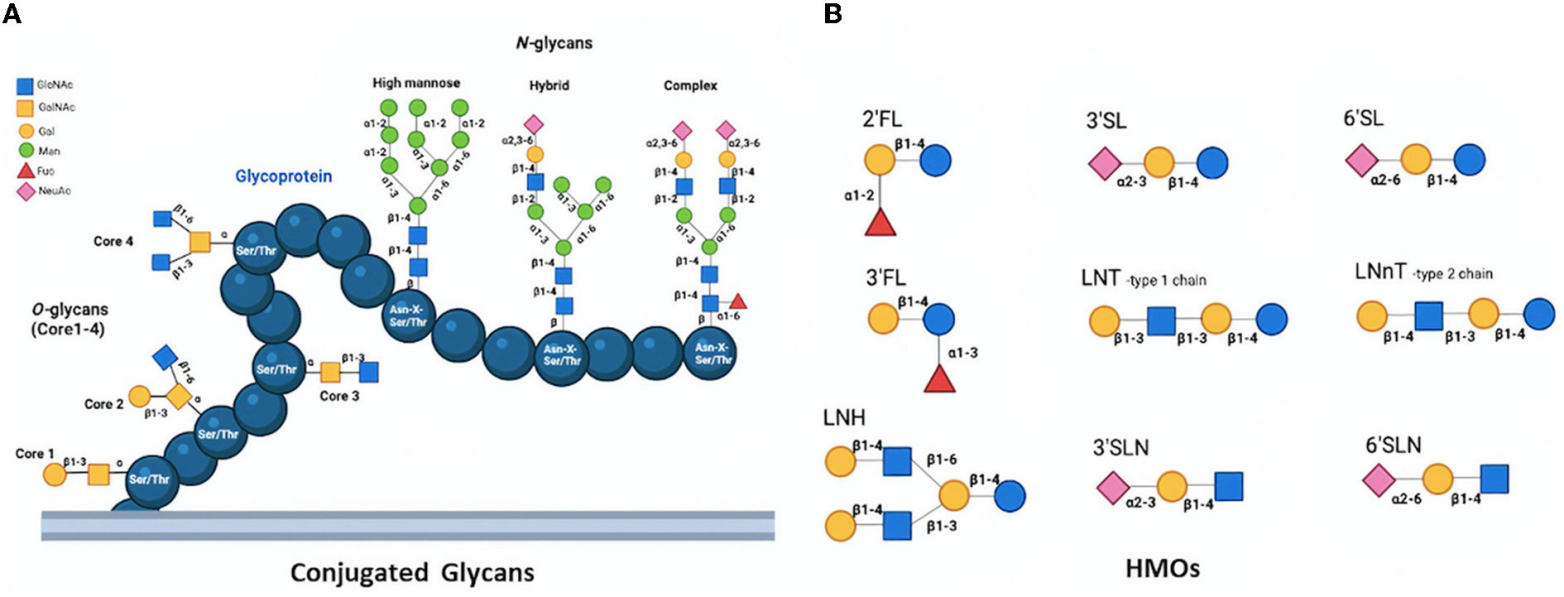
Structures of *O-*glycans (Core 1-4) and *N-*glycans (high mannose, hybrid, and complex) conjugated a glycoprotein **(A)** and Basic HMOs **(B)** (18, 19).

The protein glycosylation is the most prevalent and significant posttranslational modification and takes place *via* the conjugation of distinct sugar moieties to proteins. This modification results in a microheterogeneity of glycoproteins that influences a myriad of attributes ranging from cell-to-cell communication to immune recognition (15, 16). *N-*glycosylation and *O-*glycosylation are two main types of glycosylation in eukaryotes. While *N-*linked glycans (*N-glycans*) covalently bind to proteins at the carboxamide group in asparagine (Asn) side chain residue of Asn-X-Ser/Thr sequons, *O-*linked glycans (*O-glycans*) attach to the -OH group at the side chain of serine (Ser) or threonine (Thr) amino acids (17). *N*-glycans can be released from milk peptide chains by distinct methods and they are found in three distinct forms; high mannose, hybrid type, and complex type based on their monosaccharide sequence and branching (18, 19). Though all three types include the same core structure, the high mannose type contains only mannose (Man) residues conjugated to the core whereas the hybrid type consists of two branches; one terminates in Man and the other terminates in the sugar of complex form, and the complex type includes outer chains of sialic acid (Neu5Ac), galactose (Gal), *N-*acetylglucosamine (GlcNAc) residues, as well as α-linked Man substituted at C-2 and−6 (20, 21). On the other hand, *O-*glycans have eight different core structures whose cores 1–4 may be considered common among others (Figure 1A). Core 1 *O-*glycan structure is formed *via* the attachment of Gal to the GalNAc, whilst core 2 utilizes core 1 by introducing the GlcNAc. Furthermore, the structure of Core 3 is formed with the linking of a GlcNAc to the antigen of TN, which can be extended with GlcNAc in order to produce the core 4 structure (22). Conjugated glycans on proteins are involved in several biological mechanisms including protein folding, cell-cell or cell-host interaction, antimicrobial, antiviral, and prebiotic effects (23, 24). Over 70% of the proteins in mammalian milk are found in the glycosylated form, which can be categorized into three groups namely whey, casein, and milk fat globule membrane (MFGM) (25, 26). All three groups take critical roles in the defense system and disease prevention with their varying degrees of antiviral activity (27, 28) (Table 1).

**Table 1:** Some antiviral milk glycoproteins.

**Glycoprotein/Source**	**Virus**	**Reference**
Lactoferrin/whey	SARS-CoV-2	(9, 10, 37, 39, 42)
	HIV	(138)
	Hepatitis C virus	(139)
	Hepatitis B virus	(140)
	Human rotavirus	(141)
	Poliovirus	(142)
	Hantavirus	(143)
	Adenovirus	(144)
	Herpes simplex virus 1/2	(145)
	Influenza virus A (H1N1)	(146)
Lactoferrin and Lactoferricin/whey	Papillomavirus	(147)
	Echovirus	(148)
	Herpes simplex virus 1/2	(149)
Lysozyme/whey	SARS-CoV-2	(48, 49)
	Herpes simplex virus type I	(44)
	HIV	(150)
Lactoperoxidase/whey	SARS-CoV-2	(42)
	Herpes simplex virus 1	(151)
	A/H1N1	(152)
	Influenza virus	(152, 153)
Beta-lactoglobulin/whey	HIV	(154)
	Influenza virus A	(155)
	Human cytomegalovirus	(156)
	Papillomavirus	(157)
	Avian influenza virus (H5N1)	(158)
	Human rotavirus	(45)
Alpha-lactoglobulin/whey	Herpes simplex virus 1/2	(44)
	HIV	(158)
	Human cytomegalovirus	(156, 158)
Casein	SARS-CoV-2	(58)
Serum albumin/whey	Sindbis virus	(45, 156)
	Semlike forest virus	(13, 45)
Mucin/MFGM	SARS-CoV-2	(66)
	Rotavirus	(13)
	Norwalk virus	(72)
	Norovirus	(72)
	HIV	(63)
	Poxvirus	(159, 160)
Lactadherin/MFGM	Rotavirus	(68)
BSSL/MFGM	HIV-1	(71)
	Norwalk virus	(72)

### Whey proteins

Whey proteins account for about 20% of proteins found in milk; however, their exact content and ratio to caseins vary based on the lactation stage and species (29). Whey proteins consist of a remarkable glycoprotein content including lactoferrin, α-lactalbumin, serum albumin, lysozyme, lactoperoxidase, sIgA, and other immunoglobulins (30). Bovine milk has similar content but also contains a high concentration of β-lactoglobulin, which is quite different from human milk. Whey glycoproteins not only contain essential amino acids but also take significant roles in various biological processes including promotion of muscle strength and bone growth, reducing cholesterol, and improvement of cognitive ability. They display immunomodulatory, antimicrobial, and anti-inflammatory functions (31, 32). As for antiviral properties, whey glycoproteins are considered potential therapeutics owing to their pharmacological activities against virus-related infections (12). A variety of target viruses including influenza virus A, HIV and rotavirus can be affected by whey glycoproteins (28, 33–35).

Lactoferrin, which is an 80 kDa milk glycoprotein found in whey, has been extensively studied for its antimicrobial activity (36, 37) and considered an antiviral agent against adenovirus, enterovirus 71, human papillomavirus, rotavirus, HIV, and SARS-CoV-2 (10, 28, 36). Lactoferrin can exert an antiviral impact in different ways including direct interaction with the virus and cellular receptors on cells and stimulating immunity. Lactoferrin derived peptides (lactoferricin, lactoferrampin) also show antiviral activity against various virus types (28, 37).

The antiviral activity of lactoferrin against SARS-COV pseudovirus was shown by inhibition of viral entry in the mechanism of heparan sulfate binding in the literature previously (9, 37). Such studies on SARS pseudovirus-infected HEK293E/ACE2-Myc cells provided certain understading for lactoferrin binding to the surface of cell at heparan sulfate proteoglycans, which inhibits the binding of spike protein to the cell surface (9). Lactoferrin was also show to be able to directly attach to sialic acid residues, thus hindering the viral attachment to the cell (38). Furthermore, bovine and human lactoferrin are showed to be able to effectively block the entry of SARS-CoV-2 and other coronaviruses in different cell lines such as Calu-3, 293 T-ACE2 with the mechanism of inhibition of the host attachment through membrane-bound heparan sulfate proteoglycans (HSPGs). Bovine lactoferrin is also shown to be able to block SARS-CoV-2 replication and the production of virions when lactoferrin was introduced before the virus entry (39). In addition to the HSPG binding mechanism, other possible mechanisms have also been studied to increase the understanding of the exact interaction between SARS-CoV-2 and lactoferrin. Lactoferrin can directly bind to SARS-CoV-2, which prevents the attachment of the virus to the ACE2 receptor.

Another known antiviral mechanism of lactoferrin is related to intracellular signaling pathways (11). Lactoferrin can induce α and β interferon (IFN) with cell signaling and thus block the replication of the virus after its entry into the cell (40). It can also induce anti-inflammatory and proinflammatory cytokines such as interleukin 6 (IL6), toll-like receptor 3 (TLR3), TLR7, and interferon regulatory factor 7 (IRF7) in Caco-2 cells considerably thus increase the antiviral immune response (41). Considering clinical studies related to lactoferrin antiviral activity against SARS-CoV-2, lactoferrin could be considered a significant therapeutic agent for the prevention and therapy of COVID-19. Orally administered liposomal lactoferrin and zinc mixtures are known to result in a complete and prompter recovery from COVID-19 for all treated patients in comparison to untreated controls within the first 5 days of treatment (42). In a similar study with liposomal lactoferrin and zinc mixtures, the treatment was also found to be effective to prevent disease in treated individuals (10). Moreover, in asymptomatic, paucisymptomatic, and moderate symptomatic patients, the time needed for SARS-CoV-2 negativization in patients orally treated with lactoferrin was reported to be significantly lower than in control groups (42).

In addition to lactoferrin, other whey glycoproteins also exhibit strong antiviral activities against several viruses. For example, human and bovine α- and β-lactalbumin demonstrate high antiviral activity against some viruses such as HIV-1 by inhibiting the viral replication (43–45). Serum albumin known to have an antiviral impact against the sindbis and the semliki forest viruses by inhibiting the virus receptors on the cell surface (13). Similarly, lysozyme is also a milk glycoprotein that has crucial biological functions in protecting the host against infections. Its antiviral activity is linked with its cationic characteristics which enables lysozyme to easily attach to negatively charged membrane structure (12). Lysozyme is known to be effective against herpes simplex, HIV-1, and herpes zoster viruses (46, 47). Although the interaction between lysozyme and SARS-CoV-2 has not been studied, lysozyme aerosol treatment is known to be effective in decreasing inflammation, which could help against COVID-19-related lung complications (48, 49). Lysozyme is also known to have neuroprotective functions which can be effective to prevent neurological COVID-19 outcomes. In a study, lysozyme in combination with niclosamide has reported to decrease the lung viral load in SARS-CoV infected mice considerably (49). Lactoperoxidase enzyme is another whey glycoprotein, which belongs to the heme peroxidase family. It is known to exert a strong antimicrobial activity by interacting with thiocyanate in the presence of hydrogen peroxide to produce an antimicrobial hypothiocyanite anion. With this mechanism, lactoperoxidase inhibits many viruses including HIV, HSV-1, respiratory syncytial virus (RSV), and influenza virus. Antimicrobial anion produced by the lactoperoxidase was shown to be effective against SARS-CoV-2 at a micromolar level previously (42).

Additional to these proteins, whey immunoglobulins (sIgA, IgA, IgG, IgM, IgE, and IgD) are also involved in antimicrobial mechanisms against infections. Studies demonstrated that milk immunoglobulins of raw milk from non-immunized bovines, cows, and camels exert specific antibodies against human rotavirus (50). In addition, these molecules also known to inhibit the replication of rotaviruses in tissue culture and prevent mice from rotavirus infection. When cows are vaccinated against viral infection before taking their milk samples, IgGs from the super immune bovine shown to attach themselves to the virus directly and prevent the viral attachment to epithelial cells (51). As for SARS-CoV-2, the variety of IgG milk samples against the virus may rise with the consumption of hyper-immune milk from vaccinated cows and in turn, they could be protective against COVID-19. In a study, a human polyclonal IgG antibody derived from vaccinated cattle against MERS is shown to be safe and consumable up to 50 mg/kg in healthy people (52). In addition to these, sIgA which is a highly glycosylated protein, is found in high concentrations in human milk. It is shown to be able to exert its antimicrobial effects against several microbes and modulates immunity and also protect the host *via* the mechanism of Fab (the antigen-binding fragment of an antibody) mediated neutralization of viruses and toxins (53).

### Casein

The casein accounts for a majority (about 80%) of the protein mass in milk and includes different forms (αs1-, αs2, β-, and κ-CN). Casein fractions are the main precursors of a myriad of biopeptides including bioactive peptides (54). They have also a critical role in immunity through the proliferation of immune cells. Many immunomodulatory peptides from casein influence macrophage activity, increase the number of antibodies and regulate cytokines' synthesis, which enables them powerful anti-infection agents against different viruses (55).

Distinct forms of casein protein have different amino acid sequences and perform different functions. Even though β-casein is the most prevalent casein form present in human milk (about 75%) it does not include known glycosylation sites. κ-casein is the major glycosylated casein that accounts for about 25% of total casein and has seven *O*-glycosylation sites which noticeably contribute to its antimicrobial activity (56). κ-casein and its derived peptides enhance the growth of some beneficial bacteria in the gut such as *Bifidobacterium infantis* and *Lactobacillus bifidus* and decrease the colonization of viral pathogens in the gut (57). Furthermore, the κ-casein fraction can bind to the virus surface spike protein to inhibit the entry of the influenza virus into the cell. In a recent *in-vitro* study, casein of goat milk was shown to be an effective potential therapeutic against COVID-19 infection (58). Casein proteins are also involved in antiviral and immune regulation functions by regulating immunity response with upregulation to enhance the inhibition of viruses and downregulation to reduce harmful conditions including sepsis (59–61).

## MFGM

MFGM, which is a component of human milk, derives from the apical plasma membrane of lactating epithelial cells. It contains glycoproteins such as mucins, lactadherin, and bile salt-stimulated lipase (BSSL). MFGM glycoproteins could be effective antiviral agents against several viruses such as HIV and rotavirus; besides, they strengthen immunity against infections (27, 62, 63).

Mucin is a significant glycoprotein and the primary component of mucus structure in the gastrointestinal and respiratory tracts. Mucin types 1 and 4 are found in human milk and demonstrated to have antiviral activity against HIV, influenza virus, and other viruses *in-vitro*. They are extracellularly located and include a membrane-bound region, a short cytoplasmic segment, as well as the highly *O-*glycan part. *O-*glycans of MFGM proteins act as decoy proteins to inhibit pathogen attachment to epithelial cells (64). Sialylic acid-containing milk mucin shown to block the replication of rotavirus in tissue culture and prevented rotavirus infection in a mouse model. Deglycosylation of the mucin causes the loss of antiviral activity, the inhibition mechanism against viruses is mainly attributed to *O*-glycans in MFGM mucin (27). In addition, maternal HIV-1 transmission through the child could be prevented by MUC1 of human milk by binding to dendritic cell-specific intercellular adhesion molecule-2-grabbing non-integrin (DC-SIGN) receptors on dendritic cells which blocks the gp129 protein initiating HIV infection (65).

Recently, it has been shown that bovine mucins can inhibit infection of human coronavirus OC43 by depending on both concentration and glycan manner (66). Regarding SARS-CoV-2 targets, protection by mucins has also been possible against this virus. Furthermore, the mucin content of human milk and its viscosity can rise when the mother gets infected by SARS-CoV-2, in turn, this could be an effective protection for the newborn. Lactadherin, which is a 46 kDa glycoprotein, is a mucin-associated sialic acid-containing glycoprotein in the MFGM and includes five *N-*linked glycosylation sites (67). Lactadherin is mainly linked with the inhibition activity against rotavirus infection (8, 27). The attachment of lactadherin to rotavirus was reported to reduce after hydrolysis of sialic acid which indicates that lactadherin is a sialic acid determinant in the interaction. In a clinical report, rotavirus infection was monitored for 200 infants and compared with the lactadherin level in their mother's milk. The infected infants who were fed milk with a high concentration of lactadherin remained asymptomatic (68). However, infants fed with a low concentration of lactadherin suffered severe diarrhea. BSSL, another glycoprotein in MFGM, is responsible for fat digestion (69). BSSL is secreted from the pancreas and stimulated by bile salts in the intestine. This unique enzyme is highly glycosylated with a mucin-like C-terminal region and contains 10 *O-*linked glycosylation sites (70). With its glycans, BSSL exerts an antiviral impact on some viruses such as norovirus and HIV-1 (71, 72).

## Conjugated glycans

The most significant properties of milk glycoconjugates are attributed to their conjugate glycan parts. Since conjugated milk glycans are considerably similar to HMOs in terms of their linkages and monosaccharide composition, they play a range of biological activities (23, 24). One of the important properties of conjugated glycans is their prebiotic effects that selectively induce the growth of some beneficial microorganisms depending on glycosidase enzyme capability which might be valued in different applications (73). For instance, *N-*glycans released from bovine and human milk, exert a bifidogenic effect on specific kinds of bifidobacterial species. While *N-*glycans released from glycoproteins of bovine milk stimulate the growth of *B. infantis*, selectively which is derived from the infant's gut, *Bifidobacterium animalis* (*B. animalis*) cannot utilize these structures (24). An *in-vivo* study has shown that 19 distinct *N-*glycan structures from lactoferrin and immunoglobulins enhance *Bifidobacterium longum subsp. infantis* (*B. infantis)* growth. The study has also indicated that an enzyme produced by *B. infantis*, endo-ß*-N*-acetylglucosaminidase (EndoBI-1), can release about 800 mg of *N-*glycans from ten grams of either bovine or human milk glycoproteins (74). Previous studies regarding this unique enzyme suggested that up to 4–8% of glycoproteins including lactoferrin can be released as glycans by the activity of EndoBI-1 (74–77). In addition to EndoBI-1, recombinant bifidobacterial enzymes are highly active on conjugated *N-*glycans release (78). *N-*glycans are also fermented to short-chain fatty acids (SCFAs) like HMOs, which lowers the environment pH in the gut as well as creates a high resistance to pathogen colonization through mucin structures since they generally grow at pH 6-7. Some conjugated glycans such as sIgA and lactoferrin take the role of binding epitopes to inhibit pathogen adhesion in a similar way to soluble oligosaccharides (79–81). Therefore, conjugated glycans significantly shape the gut microbiota by providing colonization resistance, reducing virulence factors, and inflammation (82). Conjugated glycans can also play a critical role in different biological reactions to inhibit some viruses. For example, sialylation in conjugated glycans is known to affect the antiviral mechanisms positively since the sialic acid in the terminal of glycans can bind to viruses. Avian influenza viruses, major contributors to human influenza, preferentially identify Siaα2- 3Galactose (Gal)-linked receptors. In addition, sialic acid moieties on bovine milk glycoconjugates act as competitive substrates in order to inhibit viral adhesion to the receptors on the cell surface.

As the glycoconjugates are found in different concentrations in human and bovine milk, they confer distinct levels of protection. For example, a study has reported that Le^b^ blood group antigen including fucose at the terminal of human milk κ-casein inhibited *H. pylori* adhesion to stomach cells more efficiently than κ-casein isolated from bovine milk that does not include fucose antigen (81, 83). On the other hand, the high level of sialic acid in bovine milk may better help in the inhibition of other pathogens based on the structure of the receptor. High molecular mass mucin-like components isolated from bovine milk inhibited hemagglutination of *H. pylori* with its sialic acid moieties (83). Bovine milk glycoconjugates consist of high sialylation and low fucosylation such as 68% sialylation, 31% fucosylation as well as 10% high mannose. The level of sialylation and fucosylation is highest in colostrum and differs in sialylation linked with glycosylation of immunoglobulins (81). Furthermore, the bovine milk glycoconjugates are present in a higher abundance than the bovine milk oligosaccharide, which makes them considerable research interest regarding their glycan structures and antipathogenic functions.

In a recent study, *N-*linked glycoproteins derived from human and bovine colostrum as well as mature milk were compared in whey and MFGM proteins. Great numbers of diverse *N-*glycoproteins were characterized from human colostrum (68 types), human milk (58 types), bovine colostrum (100 types), and bovine milk (98 types) (84). One type of *N*-glycosite was reported to be dominant for each sample and the difference between bovine and human milk was significant with only a minority of overlapping glycoproteins. In MFGM glycoproteins' analysis, the number of types of glycoproteins was higher than whey as 465, 423, 334, and 175 for human colostrum, human milk, bovine colostrum, and bovine milk, respectively (85). Similarly, many *N-*glycoproteins from MFGM included a single site, and differences were noticed between two types of milk, probably associated with immune-related glycoproteins which vary according to the lactation periods (86). The protein content of mammalian milk is found at the maximum level in colostrum. Both bovine and human colostrum comprise the maximum concentration of bioactive glycoproteins and their conjugated glycans. For instance, bovine colostrum includes the greatest level of high glycosylated proteins such as IgGs (20–200 mg/mL) and lactoferrin (1.5–5 mg/mL) which decreases gradually by transforming through the mature milk (87). Human colostrum consists of 5–7 mg/mL lactoferrin and 1–20 mg/mL IgGs and these levels considerably decline up to 1 mg/mL in mature milk (88–90). These increased glycoproteins and their conjugated glycan levels in mammalian colostrum in comparison to mature milk is strongly associated with their greater bioactive and antiviral functions than milk (91). To date, many studies are significantly interested in how colostrum can be used as a supportive intake to benefit human health (7, 10, 92).

## Free glycans

### HMOs and their antiviral properties

HMOs are multifunctional and complex carbohydrates found in both human and bovine milk. Since the bovine colostrum consists of HMOs in high amounts, it is generally prepared to be ready for human consumption as well as health (93–96). The concentration of HMOs in human milk, in particular, varies between 5–20 g/L depending on maternity genetics and lactation level. They are the third-largest component of human milk exerting various beneficial effects on infants (23, 96). The fundamental core structure of HMOs contains lactose at the reducing end, which can be elongated by adding *N*-acetyllactoseamine units *via* glycosyltransferase enzymes in the mammary gland. D-glucose (Glc), D-galactose (Gal), GlcNAc, L-fucose (Fuc), and NeuAc are basic building blocks of HMOs (Figure 1B) (97). The chain length is between 3–15 carbohydrate units; besides, HMOs can be the linear or branched form which creates a diversity (98, 99). Based on their chemical content, HMOs categorize in three subgroups including neutral *N-*containing (non*-*fucosylated) (42–55%), neutral (fucosylated) (35–50%), as well as acidic (sialylated) (12–14%) (18). The lactose core structure can be extended with repeats of lacto-*N-*biose (Galβ1-3GlcNAc; LNB) which are named type 1 chains. Lacto-N-tetraose (LNT) as type 1 is the most prevalent HMO (100). A type 2 chain is formed when the *N-*acetyllactosamine unit (LacNAc; Galβ1-4GlcNAc) is linked to the lactose. Both type 1 and 2 chains can be further elongated by adding fucosyl and sialyl residues in order to create more diverse and larger HMOs (101). HMOs are involved in several biological functions (98, 102). They act as defensive agents against different pathogens by exerting their antimicrobial capabilities (103, 104). HMOs also act as receptor decoys, immunomodulatory agents, prebiotics, as well as signaling agents to prevent viral infections through distinct mechanisms.

#### Receptor decoys

One of the antiviral mechanisms of HMOs is acting as soluble decoy receptors or competitive inhibitors to inhibit viral attachment and entry through the host cell (32, 105, 106). Two major mechanisms related to receptor decoying were proposed to explain how HMOs cause viral inhibition in cells. Firstly, since they have a similar structure to mucin glycans found on the mucosal layer, HMOs act as soluble decoys and bind to viruses, therefore, prevent early cellular attachment of viruses. Secondly, HMOs can bind the receptors on the epithelial cell surface to inhibit viral binding, which is critical for the prevention of viral infection initiation (107). Several HMO types including neutral, fucosylated, and sialylated shown to have antiviral effects on various viruses such as noroviruses (107), rotavirus (108, 109), HIV (110), and influenza virus (111, 112). Furthermore, the similarity between HMOs and receptor glycans enables them great decoys for viruses. For example, human norovirus needs the attachment to cell surface histo-blood group antigen (HBGAs), which consists of several glycans, for viral binding to epithelial cells. Similarly, SARS-CoV-2 initiates infection by binding to ACE2 receptors on the cell surface which are highly glycosylated with mainly fucosylated glycans (113, 114). An *in-vitro* study demonstrated that A-type HBGA co-localized at the cell surface with transfected SARS-CoV spike proteins. There was less interaction between ACE2 and spike protein of SARS-CoV-2 when there were anti-A-bodies (113). Moreover, some fucosylated HMOs including 2'FL, 3FL, and LNFP 1 are structurally similar to HBGAs and can block infections of norovirus by competitively attaching to capsid protein or P domain (114, 115). Importantly, 2'FL as a fucosylated HMO may inhibit SARS-CoV-2 entry to the cell with a competitive binding (14).

#### Immunomodulatory agents

A variety of pathogens causing serious infections are identified by recognition receptors in the human immune system. Viral surface lectins, for example, recognize the glycans bound to the epithelial cell surface to identify the host when there is an infection emergency (14). For mucosal and systemic immunomodulation, HMOs can attach to lectins or glycan-binding proteins that are expressed on a variety of cells. These complex carbohydrates show immunomodulatory as well as anti-inflammatory impacts *via* unique mechanisms. HMOs have the ability to bind to the lectins directly on the surface of immune cells, promoting T cell proliferation, differentiation, cytokine production (116, 117) and dendritic cells (118, 119) involved in the anti-inflammatory mechanism. Additionally, HMOs can interact with type I interferon which is a part of the immunity mechanism against viruses. Type I interferon interacts with various immunity receptors and cells including IFN α receptors 1-2, STAT 1-2, and IFN regulatory factor 9 (IRF9) and IFN stimulated genes (ISGs) to inhibit viral replication. Human milk enhances the type I interferon formation in infants with the influenza virus, which can be caused by HMOs' contribution to the triggering type I interferon production in infected cells (120). Thus, HMOs are known to exert immunomodulatory functions by decreasing virus-related infection caused by many viruses including RSV, rotavirus, HIV, norovirus, and maybe SARS-CoV-2 (121–123).

#### Prebiotics

HMOs enhance the growth of beneficial microorganisms in the human gut with their prebiotic properties, which is an essential activity for immunity (124). As these complex molecules cannot be metabolized by human-associated enzymes, they can reach the colon in intact form and are utilized by some beneficial microorganisms such as Bifidobacteria (125, 126). As end-products of fermentation of these non-digestible carbohydrates in the human gut, various short-chain fatty acids called SCFAs formed from the microbiota, such as acetate and butyrate. In turn, this prebiotic activity of HMOs on gut microbiota significantly contributes to immunomodulating action. SCFAs not only provide energy for epithelial cells but also strongly affect intestinal homeostasis and exhibit immunomodulatory impacts (126). On the other hand, when the number of pathogens increases which produces more toxic products, gut dysbiosis takes place and the balance of gut microbiota is thoroughly destroyed. Many COVID-19 patients exhibit dysbiosis which can persist even after infection resolution (127, 128). For up to 30 days following viral clearance, commensal bacteria such as *Faecalibacterium, Eubacterium*, and *Bifidobacterium* species showed a relative decrease in the fecal samples of COVID-19 patients in a recent study (129). As altered microbiota could play a crucial role in the regulation of immunity, most of the COVID-19 patients' immunity diminished regarding gut dysbiosis. Regarding therapeutics, prebiotic treatment is one of the effective solutions that should be implemented to treat gut dysbiosis and strength immunity by supporting the growth of beneficial microbiota (129). Since HMOs are great prebiotic compounds, oral supplements of these complex and undigestible molecules have a high potential for COVID-19 infections treatments. With the supplementation of HMOs, they are fermented by healthy gut microbiota, which in turn, produce SCFA molecules that benefit intestinal homeostasis and immunomodulation (130, 131).

#### Signaling agents

HMOs can act as signaling agents to modulate various types of signaling pathways in cells. They modulate mucosal signaling cascades such as toll-like receptor 4 (TLR4) and alter epithelial cell gene expression, which improves tight junction function, lung damage, maturation in intestinal cells, and tissue repair (14). The development of epithelial glycocalyx which supports the colonization of bacteria and mucosal barrier function is also enhanced by HMOs. For instance, 2'FL and 3FL modulate the formation of the intestinal glycocalyx layer, preventing the adhesion of pathogens to the epithelial cells (115, 132–136). 2'FL, furthermore, affects the CD14 expression, which is the coreceptor of TLR4, enhancing the antagonistic effects against TLR4-mediated mucosal inflammation (137). As for SARS-CoV-2, its spike protein can also bind to TLR4 for the mediation of respiratory mucosal inflammation and lung injury. Therefore, targeting TLR4 signaling has been considered a new therapeutic for COVID-19 patients. HMOs as a mixture or separate form could be used as treatments for COVID-19 patients, especially for acute lung damage and respiratory mucosal inflammation (136).

## Conclusion

Glycosylated proteins are one of the most important components of both mature milk and colostrum and they actively take significant roles in different biological functions ranging from immunity to antiviral mechanisms. The most considerable functions of glycoconjugates are attributed to their glycan parts found in a conjugated form on protein structures. As the conjugated glycans are similar to free oligosaccharides regarding their structure and monosaccharides, they can also act as free ones in distinct biological mechanisms against viruses. Conjugated glycans, for instance, can shape the gut microbiome by enhancing the growth of beneficial bacteria, even more they can inhibit pathogens including different viruses such as norovirus, adenovirus, HIV, etc. by using unique antiviral mechanisms. Both human and bovine milk include incredible glycoprotein content ranging from lactoferrin to lactoperoxidase which exerts antiviral effects against diverse types of viruses as well as SARS-CoV-2. To date, the epidemic of COVID-19 and other potential coming outbreaks have indicated that searching for novel treatments against pathogens is critical to deal with them. As for especially human milk glycoproteins and their milk conjugated glycans, they are considered potential therapeutics to combat serious outbreaks that human beings face because of their antiviral properties. The mechanism and effectiveness of human milk on SARS-CoV-2 will be better understood by increasing the number of research and clinical applications of human milk and its components including HMOs and glycoconjugates. Importantly, glycan-rich milk, particularly colostrum, which contains a higher concentration of conjugated glycans than mature milk, may be used as a natural alternative to conventional drugs to prevent and/or treat viral diseases such as COVID-19 threatening the health of the general human population.

Regarding potential studies in the future, plant-based glycans which are highly complex and heterogeneous could be also another antiviral agent in addition to milk-based glycans. Furthermore, plants' glycoproteins and their highly variable plant-derived glycans may be used as therapeutic proteins in medicine. Consequently, the comprehension of the structures and biological functions of a variety of conjugated glycans produced from different hosts is a critical requirement to develop novel glycoprotein-based therapeutics for the treatment of viral diseases including COVID-19.

## Author contributions

SK organized the general content of the paper. MK was responsible for general editing and organizing the authors as well as the contribution for two sections. AS, SS, and HD contributed one section of the paper. AA and BP were responsible for editing and organizing the paper. All authors contributed to the article and approved the submitted version.

## Funding

SK has received funding from Uluova Süt Ticaret A.Ş. (Uluova Milk Trading Co.). The funder was not involved in the study design, collection, analysis, interpretation of data, the writing of this article or the decision to submit it for publication.

## Conflict of interest

The authors declare that the research was conducted in the absence of any commercial or financial relationships that could be construed as a potential conflict of interest.

## Publisher's note

All claims expressed in this article are solely those of the authors and do not necessarily represent those of their affiliated organizations, or those of the publisher, the editors and the reviewers. Any product that may be evaluated in this article, or claim that may be made by its manufacturer, is not guaranteed or endorsed by the publisher.
